# The evolution of uncertainty in second opinions about prostate cancer treatment

**DOI:** 10.1111/hex.12566

**Published:** 2017-05-18

**Authors:** Marij A. Hillen, Caitlin M. Gutheil, Ellen M. A. Smets, Moritz Hansen, Terrence M. Kungel, Tania D. Strout, Paul K. J. Han

**Affiliations:** ^1^ Department of Medical Psychology Academic Medical Center University of Amsterdam Amsterdam The Netherlands; ^2^ Center for Outcomes Research and Evaluation Maine Medical Center Research Institute Portland ME USA; ^3^ Division of Urology Genitourinary Cancer Program Maine Medical Center Portland ME USA; ^4^ Maine Coalition to Fight Prostate Cancer Augusta ME USA; ^5^ Department of Emergency Medicine Maine Medical Center Portland ME USA

**Keywords:** medical decision making, physician‐patient relations, prostate cancer, referral and consultation, second opinions, uncertainty

## Abstract

**Background:**

People who have cancer increasingly seek second opinions. Yet, we know little about what motivates patients to seek them and how beneficial they are. Uncertainty—experienced by patients or communicated by physician and patient—may be crucial throughout the second opinion process.

**Objective:**

This study sought to investigate (1) how uncertainty influences men with prostate cancer to seek second opinions and (2) how second opinions may affect these patients’ sense of uncertainty and subsequent experiences with their care.

**Methods:**

A qualitative study using semi‐structured interviews was performed. Men with localized or advanced prostate cancer (n=23) were interviewed by telephone about their motivations and experiences with seeking second opinions and the uncertainties they experienced. Analysis was performed using the constant comparative method.

**Results:**

Patients sought second opinions because they were uncertain about receiving too little or biased information, experienced insufficient support in coming to a treatment decision, or because physicians expressed different levels of uncertainty than they did (“unshared uncertainty”). Uncertainty was reduced by the second opinion process for most patients, whereas for others, it increased or was sustained. This evolution depended on the way uncertainty was addressed during the second opinion consultation.

**Conclusions:**

Second opinions may be a useful tool for some but not all patients. They should be used judiciously and not be viewed as a solution for current limitations to health‐care organization. An important yet challenging task for physicians is to focus less on information per se and more on how to assist patients manage irreducible uncertainty.

## 1 INTRODUCTION

When confronted with a serious disease, patients may request a second opinion: soliciting the assessment of a diagnosis or treatment proposal by a second, independent physician about the same clinical condition (adapted from^1^, ^2^). Patients are increasingly pursuing second opinions, although precise data on their frequency are lacking.^3^ For people who have cancer, the high‐stakes nature of diagnosis, prognosis and treatment heightens the significance of the uncertainties that surround these aspects of care. Patients’ experience of these uncertainties may be an important element causing patients with cancer to seek second opinions. Indeed, limited evidence thus far suggests that between 5 and 36% of patients in oncology have sought a second opinion.^4^

In some settings within oncology, seeking a second opinion has become more a matter of course than an exception, because multiple, medically equivalent treatment options exist. In such situations, patients’ values and preferences should be taken into account to arrive at an individualized treatment decision. Ideally, a single health professional would inform patients in such situations about all available treatment options and assist them in arriving at a decision.^5^ In some settings, however, health professionals may inform patients only about the option(s) within their field of expertise and encourage second opinion seeking to collect information about other treatments. For example, men who have prostate cancer are frequently expected or encouraged to pursue multiple opinions from different specialists to be fully informed about all available options.^6^, ^7^, ^8^

Seeking a second opinion may offer several possible benefits to patients. Psychologically, it can reduce anxiety or increase their sense of control. Medically, it may lead to a more precise diagnosis or better care.^9^, ^10^, ^11^ On the other hand, the vast majority of second opinions appear not to benefit patients medically.^10^, ^12^, ^13^ Moreover, they may slow down the diagnostic process and be physically and financially demanding for both patients and physicians.^9^, ^11^, ^13^ The potential harms and benefits of second opinions have created debate about how desirable these consultations are.^11^, ^14^

This debate is impaired by limited insight on people's motivations to seek second opinions, how they evolve and their consequences. Uncertainty may be a crucial element throughout these different phases of the second opinion process. Uncertainty, defined as a “subjective perception of ignorance”,^15^ has long been acknowledged as central to medical practice.^15^, ^16^, ^17^ Patients’ uncertainty may pertain to numerous issues, including the accuracy of their diagnosis, the efficacy of their treatments, their prognosis and the quality of their care. Uncertainty—as experienced by patients, physicians or communicated between physicians and patients—may influence patients’ decision to request a second opinion. Moreover, patients’ levels of experienced uncertainty may be influenced by the second opinion, and how this uncertainty changes may ultimately affect patients’ care experiences and outcomes.

Currently, we lack empirical evidence and in‐depth understanding of the role and evolution of uncertainty in second opinions. In this study, this problem was investigated in the prostate cancer setting, where uncertainty is abundant. Clinically localized prostate cancer is an exemplary “preference‐based medical condition”: none of the available treatment options, that is radiotherapy, surgery and active surveillance, are medically superior in terms of potential benefits and harms for individual patients.^18^ Substantial uncertainty also exists regarding the optimal treatments for advanced prostate cancer, that is hormonal, radiation or chemotherapy. Patients with both localized and advanced prostate cancer may seek second opinions to deal with these uncertainties. Other issues of uncertainty may also be important to patients with prostate cancer, but their nature remains to be identified.

The aim for this study was to explore in‐depth the evolution of uncertainty in second opinions about prostate cancer, specifically (i) what specific uncertainties influence patients to seek second opinions and (ii) how second opinions may affect patients’ sense of uncertainty and subsequent experiences with their care. Findings may contribute to insights about the second opinion process and may yield strategies to help both physicians and patients in communicating about and dealing with uncertainty.

## 2 METHODS

A qualitative study using semi‐structured interviews was performed, using the constant comparative method.^19^ This method is aimed at generating theory based on the data, rather than conducting analysis based on an existing theoretical framework.^20^ Analysis using the constant comparative method begins with open coding (summarizing and categorizing the data), followed gradually by axial coding (confirming codes and examining broader relationships), resulting in the identification of common themes.^21^

### 2.1 Participants and recruitment procedures

Patients with prostate cancer across the disease spectrum (clinically localized through metastatic) who requested a second opinion were purposefully recruited through two channels. First, at Maine Medical Partners—Urology (MMPU), an eight‐physician urology practice in Portland, Maine, patients who either visited for a self‐initiated second opinion or requested to be referred for a second opinion to a different institution. Second, patients visiting support groups run by the Maine Coalition to Fight Prostate Cancer (MCFPC), a statewide patient support and advocacy organization, were invited to participate if they had self‐requested a second opinion in the past 2 years. Furthermore, patients were included of various ages, socio‐economic backgrounds and types of second opinions (diagnostic and therapeutic), to capture variation in these characteristics with regard to uncertainty experience. Participants were rewarded a $50 gift card incentive. The Maine Medical Center Institutional Review Board exempted the study from review.

Eligible patients were informed about the study face‐to‐face or by telephone by either a patient navigator (MMPU) or by a support group moderator (MCFPC). Potential participants provided verbal initial consent to be contacted. The researcher next contacted patients by phone, providing additional information about the study, answering questions, requesting definitive consent and scheduling the interview. Recruitment and data acquisition stopped when three consecutive interviews did not yield any new information.

### 2.2 Data collection

In‐depth, semi‐structured telephone interviews were conducted and audio‐recorded by an experienced interviewer (MH) with a background in psychology. The interview protocol is displayed in Table 1, and evolved primarily around motivations and experiences of the second opinion, and the experience of uncertainty in the second opinion process. Throughout the interview, any expressions related to experiencing or communicating about uncertainty were explored in‐depth.

**Table 1:** Interview protocol

1. Introduction by the researcher: Explanation of the study purpose and procedure; explanation of voluntary participation, confidentiality and anonymity
2. Sociodemographic and disease characteristics, illness trajectory so far
3. Second opinion Reasons for requesting a second opinion Expectations of the second opinion consultation Experience of the second opinion process Outcomes of the second opinion
4. Uncertainty The sources and types of uncertainty experienced Dealing with uncertainty The role of uncertainty in the second opinion process The influence of the second opinion on uncertainty Communication with physicians about uncertainty
5. The role of trust in the second opinion
6. Conclusion: Questions and remarks, explanation of further procedure

### 2.3 Analysis

All interviews were transcribed verbatim and analysed using MAXQDA12,^22^ following guidelines for qualitative research.^23^ During data acquisition, three interviews were read in‐depth and discussed by three authors (MH, CG and PH; with backgrounds in psychology, public health, and medicine and bioethics, respectively) to allow revising of the protocol. Second, these authors independently coded and jointly discussed three other interviews to arrive at an initial coding scheme. Subsequently, two authors (CG and MH) coded all interviews independently. Codings were compared and discussed after each three consecutive interviews, with a third author (PH) present. The coding scheme was revised continuously, based on outcomes of the analysis. Initial codes were grouped per theme and then hierarchically organized. Throughout the analysis, all authors kept notes of broader themes emerging from the data. Finally, common themes related to second opinions and uncertainty were derived from clustering of the data. A fourth author (ES; background in psychology) critically reviewed general themes identified in the analysis.

## 3 RESULTS

Of the 34 patients approached for participation, 10 declined or could not participate, because they could not be reached (n=9), or felt uncomfortable with an interview (n=1). Data of one other participant were disregarded because of insufficient technical quality of the audio‐recording. Patients’ mean age was 65 years (range 52‐73). Thirteen patients had early‐stage prostate cancer, of whom six had not yet chosen a treatment option. Of the nine patients with advanced prostate cancer, six were currently receiving some form of treatment and one had not yet received any treatment. Seven patients (30%) had high school or some college, 11 (48%) had a college degree, two (9%) had a graduate degree, and three (13%) had a PhD or postgraduate degree. All patients were US‐born.

Patients’ most salient uncertainties contributing to their wish for a second opinion were [1] uncertainty about the information received—due to suboptimal timing, perceived bias or perceived insufficiency (par. 3.1.1); [2] uncertainty about how to integrate the information—due to lack of guidance or need for decision support (par. 3.1.2); and [3] uncertainty about the physician's alignment with the patient's perspective—due to unshared uncertainty (par. 3.1.3). Seeking a second opinion reduced uncertainty for the majority of patients. However, for others, it led to sustained or increased uncertainty. How patients’ uncertainty was affected depended on the extent to which their information needs (par. 3.2.1) or need for decisional guidance (par. 3.2.2) was addressed and the extent to which the physician providing the second opinion shared their level of uncertainty (par. 3.2.3). All quotes supporting the results are displayed in Table 2.

**Table 2:** Overview of quotes supporting how uncertainty causes second opinion seeking and is influenced by it

	Experience of uncertainty	Influence of uncertainty on second opinion seeking
**Uncertainty about the information received**		
Poorly timed information	**Q1**: You know [with the first urologist] we were just confused. At first he started talking about recurrence and my wife and I were sitting there looking and I said, why is he talking about recurrence? And he was talking about the different ways of attacking this and the chances of it coming back but that was like 3 steps down the road as far as I was concerned. (P003, 69 y, early stage)	**Q3**: So once I got the results [of the biopsy], this first urologist wanted to basically do surgery. He said: you know you could wait and you might live until you're 70 but I think you should do the surgery and we should do it within the next month or so. So at that point I kind of put on the brakes, and I was already not liking this guy anyway, so I pretty much told him thanks and I don't think I'll be seeing you again. (P012, 64 y, early stage)
	**Q2**: Dealing with a urologist who diagnosed me, told me about the cancer in that very same meeting, who sort of suggested that I do surgery pretty rapidly, I felt a little uncomfortable, as though I was being pushed into things just a tiny bit. But I think that was maybe because I just didn't like the fact that I just heard I had cancer and I was scared to death at the time. It was going fast and I maybe wasn't in the right frame of mind to really be receptive to him at that time. (P011, 64 y, early stage)	
Insufficient or biased information	**Q4**: The information just wasn't put out there. I mean I knew about active surveillance, the hormone, the radiation; it just, I don't know, nothing was really explained to me as well as Dr. A [who provided the second opinion] explained it. [The first urologist] went through the options but didn't really go deep into it. (P020, 59 y, early stage)	**Q6**: The surgeons were pushing surgery […] I liken them to a car dealership. In other words, the surgeon was the Volvo dealership and they wanted to sell Volvos and the radiation was the Mercedes dealership and they were pushing Mercedes and I just felt that there was too much conflict there for me to make a good decision at that time. And that's when I sought […] a second opinion. (P010, 72 y, early stage)
	**Q5**: The first two doctors, I really felt were trying to push me towards surgery. […] I guess that I sort of felt like if I could use the analogy that when you're a hammer, everything looks like a nail. (P012, 64 y, early stage)	**Q7**: I mean these people, as far as I can tell, they all know what they're talking about very well, and there have not been any inconsistencies in what they've told me; it's just that each person has his special area of expertise and as I've gone for more opinions, I've been able to ask more and better questions myself. It's a learning process. You start out knowing nothing. If I could've asked the first guy all the questions I've thought of now, then it might've been a shorter process. But each of these people has given me the scoop on what they do and the pros and cons in a firsthand way. That's been very valuable. (P031, 68 y, early stage)
High information needs	**Q8**: There's some people who'll just go home and forget about it but I don't. So once I know the results but don't understand them, I'm going to start Googling everything having to do with prostate cancer and Gleason scores and PSA's and everything else. And you can imagine what kind of results you get. A lot of them are fairly scary and depressing. Because it's not contextualized properly. So it creates a lot of anxiety and worry and further misunderstanding. (P006, 54 y, early stage)	**Q9**: But still, it's worth the effort and I frankly was fortunate that I have the flexibility financially to go do this sort of stuff and most people don't. But I'm working part time so I did not have a time or economic restriction to pursue these things. And most people simply can't do that, and I'm well aware of that. (P021, 67 y, advanced)
		**Q10**: A dear friend of mine is a surgeon at [hospital A]. I said, look I'm in a terrible pickle; I need the best you have down there. And this guy arranged for me to see Dr. C. So I was lucky because of these contacts. At [hospital B], again I could get to a high level guy pretty quickly. […] I: And is there a moment where you have enough information? R: Well it's a matter of diminishing returns. I think if you hit two people at major centers and they seem to pretty well coincide and they seem to talk about the same body of information. [.] But if they disagreed a great deal or if there was some serious issue like that, then I wouldn't have any hesitation but to go to a third person. (P017, 72 years, advanced)
**Uncertainty about how to integrate the information**		
	**Q11**: It's like you get an appliance that's supposed to make your work easier for you but you don't know how to use it. You have the education; you don't know how to apply it. (P027, 62 y, advanced)	**Q15**: One thing in my mind is, is there someone out there who would be, in terms of the type of treatment, a neutral. That I could talk with or consult with. I'm considering maybe seeing if my urologist would be willing to have another consultation where he's kind of a neutral that I have confidence in. (P016, 69 y, early stage)
	**Q12:** I: And could the doctors play any role in reducing the uncertainty about the right choice? R: They did not. No one sat down and said, well I think you should do this. It was just another situation where they said: here are your options; you could make a case on either side; you go ahead and decide. […] I had tons of information, tons of information but no guidance in like, if it were me, I would do this or I think you should do this. (P011, 64 y, early stage)	
	**Q13**: And I remember I called my urologist down in Boston. And he says, it's kind of an opinion. So I felt I was left sort of dangling there and there was nothing I could do to… and I'm certainly not, I don't have the education. To say you know, this is what should be done. (P029, 53 y, advanced)	
	**Q14**: R: The doctors are verbally helping me to decide by telling me what's involved in every procedure. So I'm going to make the choice but they're giving me the information to help me do so […] And l I feel that it's my body that's going to be affected; it's that simple. […] I need to know all the options and I need to, in order to get those I need to have professionals tell me them. (P005, 66 y, early stage)	
**Uncertainty about physician's alignment with patient's perspective**		
Physician does not share patient's uncertainty	**Q16**: I: Yeah, so how well was she able to help you deal with the uncertainties that you just mentioned about your future? R: Well I don't think she really dealt with it other than to say, we're doing the right thing right now, this is the right treatment program. And that was about it. I: Yeah and that was not enough for you at that point? R: I don't think it was. (P028, 66 y, advanced)	**Q17**: Well, that doctor thought that because my biopsy revealed so little of the cancer in the prostate that I would be the ideal candidate to take the active surveillance route. And just keep monitoring it with occasional blood tests; maybe follow up biopsies. But this is a complicated business; it's very, very complicated, you can't tell how fast the cancer is going to spread and if it'll spread, how fast, whether it will get outside the prostate. There are many things to worry about down the road so I wanted to, I got a second and also third opinion. (P031, 68 y, early stage)
		**Q18**: Although when I told her [dr. B.] that I was looking at this clinical trial, she was not particularly encouraging about pursuing that. She said she was perfectly comfortable with me just doing the surveillance. But both my wife and I decided that the opportunity to find out more by being in the clinical trial made a lot of sense to us. (P012, 64 y, early stage)
Patient does not share/tolerate physician's expressed uncertainty	**Q19**: And indeed this is true of I think most prostate cancer patients and survivors, is that there's a great desire to have an idea of what kind of future you'll have. You know 5 y, 3 y, 2 y or is it 10 y? And the doctors, it puts them on the spot and they really can't say. (P017, 72 y, advanced)	
	**Q20**: I: And so how have the doctors that you've talked to influenced your uncertainty about this whole decision? R: I don't really, you know. I don't really think they've made it worse; I think you know dr. B, my own urologist, after coming out and seeing him and everything, I think he was the most positive; you know he seemed the most reassuring to me that either way, you know the surgery or the radiation, I'm going to get to see my grandkids grow up and, you know, it's just a bump in the road. (P034, 52 y, early stage)	

	Effect of second opinions on patients’ uncertainty	
	Reduction in uncertainty	No reduction or increase in uncertainty
**Uncertainty about the information received**		
Poorly timed information	**Q23**: We had all kinds of questions because before we met with dr. B, we'd been doing some reading on cancer and we knew a lot; we were much more knowledgeable about the whole process. And he was very good about talking about, you know, what's going to, what could be happening down the road. What he's going to do, what the options were… (P003, 69 y, early stage)	
Insufficient or biased information	**Q22**: And it was just the approach that they took with the comprehensive things; so the standard of care was much different and that was one of the things that impressed me greatly. Plus the other point was that I had a meeting the same day just an hour apart with the surgeon and also the radiation doctor down there and at one point, the surgeon, right after discussing with me and my wife said, I think you'd be an excellent candidate for radiation and not surgery. And this was the first time I'd heard the Volvo dealer recommending a Mercedes. (P010, 72 y, early stage)	**Q25**: It's confusing, particularly with people at high levels. I mean, [hospital F] is so organized that you have a doctor for only a certain period of time. And it's not that they're rushing you really, but they have a lot of work to do during the day. And if you have a person for an hour, you're trying to digest things on a technical level that you're not really equipped to do. […] And that can be intimidating to the point where you're even hesitant to ask some questions. And so at the end of the hour, it is very easy to leave that appointment and not be sure of exactly what they're telling you. And that's an intimidating position for a patient to be in. (P021, 67 y, advanced)
		**Q26**: So [hospital B] was telling me one thing; kind of standard procedure protocol. And [hospital F] which I have a lot of respect for was telling me something quite different. So I was sort of confused by that, so I thought I'm going to get a third opinion. (P021, 59 y, early stage)
High information needs	**Q21**: I liked the doctor at [hospital E]; I liked his mannerisms, I liked the way he spoke to both of us, he didn't rush us, he answered all the questions and everything, and there was no pressure at all from him either. […] And I would say the third doctor I saw down at [hospital E] was very kind and you know and he was just very open and willing to spend time and everything. And it helped. I still have cancer so obviously the surgery didn't, wasn't successful. But it helped allay some of my fears about it and answered some of my questions about the surgery and the prospects and so it did help me; it helped convince me to do that. (P014, 60 y, advanced)	
**Uncertainty about how to integrate the information**		
	**Q27**: The thing that I liked about [hospital G] was that once I got the news that I needed to do something because I had a Gleason 8, their attitude was, okay well you're going to sit down with the group of physicians, like a radiation guy, a surgeon guy and an oncologist. And we're going to talk about this and nobody's going to be saying ‘you know you should definitely do this or you should definitely do this’; each doctor is going to explain what they do and the pros and cons of it. And then you know decide what you want to do. (P012, 64 y, early stage)	**Q28**: And she came in and she looked like she was like super rushed and she said, okay here's your case, what do you want to know? And I said, well in my situation, what's should I do? I'm looking for answers. I need to know what do I do from here. So she gave me a couple of options and she was very curt with me and so I left there thinking you know, wow, that was just not what I expected. And I left there with really not a whole lot of answers. It was like she said, it's your choice; what do you want to do. (P027, 62 y, advanced)
**Uncertainty about physician's alignment with patient's perspective**		
Physician does not share patient's uncertainty	**Q29**: I: And if I understand correctly, first you wanted to do the surgery but now you decided to go for active surveillance? R: Right, right because now we have a contingency plan. […] And she said one of the main things; can you live with the thought knowing you have cancer, that it's in you. (P020, 59 y, early stage)	**Q30**: Well it was a second opinion in terms of another form of treatment. I'd already had my primary treatments which failed, and so it was another opinion; I don't know if I'd call it a second opinion or a fourth opinion really. But it was really for another avenue which she basically said I did not qualify for at that point. (P004, 65 y, early stage)
	**Q31**: I: And how can your physicians help you deal with the uncertainties?	
	R: They can if they say something like: there's four medicines coming out this year, or there may be some immunotherapy's that will extend your life beyond seven years. Any chance of hope is very good […], any advancement in cancer treatment to me is a good sign. For instance, they now have an immunotherapy for lung cancer. That's a good sign you know. There's been studies of cancer, prostate cancer in mice that they can totally eliminate. So I find these things give me hope. And that's what keeps my attitude positive. (P029, 53 y, advanced)	

### 3.1 How uncertainty influences patients to seek second opinions

#### 3.1.1 Uncertainty about when, how and how much information is provided

Patients reported several ways in which their need for information was not properly addressed, causing uncertainty and leading them to seek second opinions (Table 2). First, suboptimal timing—*when* information was provided—caused uncertainty: newly diagnosed patients reported feeling overwhelmed at the time of diagnosis, when many urologists immediately started talking about treatment options. Several patients reported this was too early for them, as they were still processing their cancer diagnosis, which caused high levels of uncertainty about their life and future. This suboptimal timing led to uncertainty about the meaning of information and how to use it, as illustrated in Q1 and Q2 (Table 2). Seeking a second opinion was a means for some patients to acquire more time to process the information and make a decision, as illustrated in Q3.

A second issue causing uncertainty was *how* the information was delivered: patients perceived the information they received to be insufficient, biased or otherwise unreliable (see Q4). Patients reported feeling that the information they received was specifically incomplete because it was biased towards the physician's own specialty (eg surgery for the urologist, radiotherapy for the radiation oncologist), thereby exacerbating therapeutic uncertainty, as illustrated in Q5. Some patients reported seeking second opinions to be provided with a fuller picture of the treatment options and hence reduce their uncertainty (Q6 and Q7).

A third issue was *how much* information was provided. Some patients acknowledged having high information needs, as illustrated in Q8. For these men, uncertainty arose because their needs were not being met in their first consultation. To arrive at a sense of certainty, these men often sought multiple opinions. A few patients referred to this as “buying more certainty” and felt privileged to have the funds and connections to access a greater number of highly expert physicians (Q9 and Q10).

#### 3.1.2 Uncertainty about how to integrate the information

Other patients felt fully informed, but still experienced uncertainty about how to synthesize and use the information to come to the right treatment decision. The experienced lack of guidance caused distress, decisional uncertainty and feelings of abandonment as illustrated in Q11‐Q13 (Table 2). Patients with decisional uncertainty sought second opinions to reduce their uncertainty or acquire more neutral treatment advice (Q15). Yet although many patients perceived the lack of decisional support as negative, others reported feeling comfortable about making an individual decision (Q14).

#### 3.1.3 Uncertainty about the physician's alignment with the patient's perspective

A third way in which uncertainty played into second opinion seeking emerged when patients perceived a mismatch in the sense of uncertainty felt by them and their physicians. This occurred when patients either perceived their physician as overly certain or did not share or tolerate the uncertainty expressed by their physician.

On the one hand, some patients felt their physician was overly certain. For patients who felt that such certainty is never warranted, the physician's certainty raised suspicion and a need for confirmation. In this case, there was a mismatch between the physician's lack of expressed uncertainty and the patient's uncertainty (or lack of acceptance of certainty). Several patients indicated they would have liked to discuss their uncertainties with their doctor but felt it was not welcome or invited (see for example Q16). This mismatch in uncertainty would lead patients to seek a second opinion (Q17). A few patients reported not just experiencing, but *needing* uncertainty about possible alternative treatment options, to maintain a sense of hope. If their physicians did not acknowledge this need for uncertainty, they would similarly seek a second opinion (Q18).

Other patients, however, did not share the uncertainty expressed by their physician due, for example, to a high need for prognostic certainty that their physician could not provide (Q19). Similarly, some patients acknowledged preferring the most “reassuring” opinion that best met their need for certainty about treatment options (Q20).

### 3.2 Effects of second opinions on patients’ uncertainty

Seeking second opinions had variable effects on patients’ uncertainty. Although it reduced uncertainty for many patients, others reported that their uncertainties remained or even increased. The extent to which patients’ level of uncertainty was affected by the second opinion depended on how patients’ information needs or desire for decisional guidance was addressed, or the degree to which the physician providing the second opinion shared their level of uncertainty (see Table 2).

#### 3.2.1 Meeting patients’ need for information

In many cases, patients’ information needs were better met in the second opinion, which reduced their uncertainty. For some, the later timing of the second opinion helped them to be better prepared and, resultantly, better able to comprehend the information provided (see for example Table 2, Q23). For other patients, uncertainty was reduced because they perceived the information in the second opinion to be less biased (Q22) or better meeting their high information needs (Q21). Conversely, for other patients, the second opinion resulted in *greater* uncertainty, because they felt their information needs were still not adequately addressed. These patients reported, for example, receiving large amounts and very technical information during the second opinion, as illustrated by Q25. Others felt more uncertain because the information and advice provided in second opinion conflicted with the first opinion (Q26).

#### 3.2.2 Providing decision support

For some patients, decisional uncertainty was reduced as a result of the second opinion. These patients reported experiencing a greater sense of collaborative engagement in their second opinion: not only did they perceive the physicians to be more neutral and bias‐free, but they also felt the physicians collaborated with them in making a treatment decision, as illustrated in Q27.

Conversely, some patients’ decisional uncertainty was not reduced. They felt that, despite seeking more than one opinion, they did not receive sufficient guidance to make a decision (Q28).

#### 3.2.3 Sharing patients’ level of (un)certainty

To some patients, the perception that the physician performing the second opinion shared their uncertainty had a beneficial effect. One patient, for example, felt uncertain about the treatment option of active surveillance, but was reassured by the acknowledgement of this uncertainty by the physician performing the second opinion (Q29). Another patient expressed a need for uncertainty about the effectiveness of alternative or future treatments to retain hope and felt that an acknowledgement of this uncertainty by his physicians would allow him to maintain a positive attitude (Q31). Conversely, for others, the need to maintain uncertainty was not met by the second opinion. For example, one patient sought a second opinion to maintain a sense of uncertainty and hope about possible treatments, but found that this hope was not confirmed by the oncologist providing the second opinion (Q30).

## 4 DISCUSSION

This study explored how uncertainty experienced by men who have prostate cancer plays into second opinion seeking. For patients in this study, uncertainty was both an important motivator for and outcome of the second opinion process. Patients sought second opinions mainly because of uncertainty about the information they received, how to integrate this information in treatment decisions or the extent to which their physicians’ uncertainty aligned with their own. Uncertainty evolved throughout the second opinions process: for most patients, it was reduced, whereas for others, it increased or was sustained. This depended on the sources of uncertainty experienced by patients and how it was addressed during the second opinion consultation.

### 4.1 Adequacy of information

Uncertainty was frequently the result of patients’ perception that the information they received was incomplete or biased. They used the second opinion to obtain a better overview or validation of the initial doctor's recommendation. Patients’ information needs ranged widely: some expressed high needs and were comfortable acquiring as much information as possible, whereas others were not comfortable with too much information and felt overwhelmed by it. Patients may have different coping styles in managing threatening information. Low information seekers do not actively seek information or even avoid being confronted with it.^24^ High information seekers may actively aim to reduce their anxiety by seeking more information, even though that information may in some cases yield even more uncertainty.^25^ Patients with high information needs in the present study expressed the belief that second opinions should be routinely obtained. Some patients appeared to have more resources to actually satisfy their high information needs, which allowed them to “buy more certainty”—as second opinions can sometimes be costly—by obtaining more information from different sources. This is an important avenue for future research, as it suggests that people with lesser financial resources but high information needs might be forced to tolerate more uncertainty than people with greater resources—potentially exacerbating health disparities.

Proper timing of the information may be crucial to prevent patients from feeling uncertain and overwhelmed in processing the highly complex information provided to them directly after diagnosis. Indeed, several patients in the present study reported seeking a second opinion to buy more time to get in the right state of mind to process all the information. This suggests that the need for second opinions might be lessened if physicians could more proactively assess patients’ degree of emotional distress following disclosure of the cancer diagnosis, and adjust the timing and amount of subsequent information accordingly.

### 4.2 Adequacy of decisional support

The results further suggest that even when patients receive sufficient information, they frequently experience uncertainty because of inadequate decisional support. Information about treatment options produced uncertainty due to its volume, complexity, inconsistency and unpredictability, which patients felt they had to manage on their own. These patients sought a second opinion to resolve the feeling of being overwhelmed by having to make difficult choices by themselves. This feeling can be exacerbated when physicians do not implement truly shared decision making (SDM),^26^ which is the ideal model for the preference‐sensitive medical decisions patients are confronted with in prostate cancer. Conducting proper SDM involves introducing choice, explaining the available treatment options, helping patients explore their values and preferences and coming to a decision.^5^, ^27^ By engaging in SDM, clinicians could reduce patients’ therapeutic uncertainty. However, inadequate use of SDM could increase, rather than decrease, uncertainty. Physicians need to provide sufficient guidance to help patients make sense of the different equivalent treatment options and their associated (dis)advantages.^28^ Unfortunately, in current prostate cancer practice, SDM may sometimes be misinterpreted as a full, unilateral delegation of all decisional responsibility to the patient, rather than a mutual sharing of responsibility driven by patients’ preferred level of involvement in decision making.^8^ The organization of prostate cancer care and similar “preference‐sensitive” conditions—with individual specialists informing the patient only about their “own” treatment option (eg urologists discussing surgery exclusively and not radiation therapy)—may actually exacerbate this problem. Moreover, a lack of coordination of information between specialists could contribute to patients’ perceptions of receiving biased information from individual physicians. These perceptions can be accurate, furthermore, as physicians may, more or less consciously or unconsciously,^29^ nudge patients towards a preferred treatment direction.^8^

Multidisciplinary consultative care could reduce patients’ uncertainty, by facilitating a more collaborative approach towards decision making and a reduction in perceptions of bias.^7^, ^30^ Indeed, our data suggest that the very existence of the collaboration, independent of the information that was exchanged, may have an uncertainty‐reducing effect. Increasingly, nurse navigators have been appointed to assist patients around treatment decision making.^31^ Their involvement may provide critical decisional support, although they cannot fully replace the physicians’ role in guiding patients through the decision‐making process,^32^ and there remains a high need for different physicians to efficiently communicate with one other.

### 4.3 Unshared uncertainty

The findings on patients’ need to feel a shared level of uncertainty with their physicians may be an extension of Epstein & Street's notion of the “shared mind”^33^—the process by which two or more people create new ideas through the sharing of thoughts, feelings, perceptions, meanings and intentions. Our findings illustrate the importance of shared mind regarding patients’ level and perceptions of uncertainty. Lack of shared mind on uncertainty would drive patients to seek other opinions until they arrive at a shared level of uncertainty with their physician. This finding aligns with earlier theoretical work emphasizing how uncertainty residing in the mind of only the physician or the patient may cause conflict or distrust.^15^ Enhancing a state of “shared mind”—where the locus of uncertainty lies in the mind of both the physician and patient—may reduce patients’ need to seek multiple opinions. Encouraging physicians to be more receptive to discussions about uncertainty could promote this shared mind and foster trusting physician‐patient relationships.

This may be especially important for patients who have a high a *need for uncertainty* as a source of hope. In our study, this phenomenon was observed mainly among patients with advanced prostate cancer, who reported a desire to preserve a sense of uncertainty about the future, for example about the possibility of new treatment options. This strategy of maintaining uncertainty has been described earlier, for example in Brasher's Uncertainty Management Theory and Mishel's theory of uncertainty in chronic illness.^34^, ^35^ Patients who feel unsupported in their need to retain hope might be compelled to seek additional opinions elsewhere. Greater awareness of patients’ perceptions and needs regarding uncertainty may enable physicians to better meet these needs—and potentially reduce second opinions.^36^, ^37^

### 4.4 Recommendations

The recent increase in patient‐initiated second opinions increases financial burden on the health‐care system.^13^ The present results additionally show that because of their potentially diverse effects on patients’ well‐being, they should be used judiciously. Second opinions may be a useful tool for some but not all patients, and they should not be viewed as a solution to current limitations in the organization of care. Some sources of patients’ uncertainty could be addressed within the initial consultation, obviating the need for a second opinion. However, our results suggest that some care delivery processes are better than others in meeting patients’ needs for certainty and uncertainty. For example, offering a multidisciplinary team‐based approach to guide patients through the treatment decision may be better than having patients independently visit multiple specialists. Clinicians need to be trained in SDM to enable them to share decisional responsibility with patients, instead of delegating it completely, and to assess patients’ preferred involvement in decision making. Both clinicians and patients could also benefit from existing tools to promote decision making, such as question prompt sheets or other decision aids.^38^, ^39^ Both multidisciplinary collaboration and enhanced SDM may result in a significant reduction in uncertainty experienced by patients. Similarly, it may be better to tailor the timing of treatment discussions to individual patients’ preferences and abilities, rather to treat all patients in the same manner. These care processes, however, may be challenging to implement and require substantial changes in the organization of prostate cancer care.

Yet some of the problems that motivate patients to seek second opinions may not be addressable in any other way. Even with optimal information provision, uncertainties will always remain not only in prostate cancer care, but health care in general.^40^ Some of these uncertainties might be reducible, and this possibility—as well as the inherent challenge in determining the extent to which any given uncertainty is reducible—will motivate people to seek second opinions. These possibilities arguably justify the provision of second opinions by the health‐care system. And yet many uncertainties in health care are irreducible, and both patients and physicians must ultimately reach a point at which they must stop seeking more information.^35^ At this point, the challenging task for physicians is to focus less on information per se and more on how to assist patients in managing irreducible uncertainty. The problem of second opinions in oncology care thus ultimately comes down to the problem of helping people tolerate uncertainty.^15^ Determining exactly how to address this problem is the critical future challenge.

## CONFLICT OF INTEREST

The authors declare that they have no conflict of interest or financial relationships to report.
